# Impact of the COVID-19 pandemic on the health situation of the Brazilian population

**DOI:** 10.1016/j.bjid.2026.105790

**Published:** 2026-02-26

**Authors:** Célia Landmann Szwarcwald, Wanessa da Silva de Almeida, Paulo Roberto Borges de Souza Junior, Euclides Ayres de Castilho, Giseli Nogueira Damacena, Crizian Saar Gomes, Deborah Carvalho Malta

**Affiliations:** a Fundação Oswaldo Cruz (FIOCRUZ), Institute of Scientific and Technological Communication and Information in Health, Rio de Janeiro, RJ, Brazil; b Universidade de São Paulo, Faculdade de Medicina, Department of Preventive Medicine, São Paulo, RJ, Brazil; c Universidade Federal de Minas Gerais, School of Nursing, Belo Horizonte, MG, Brazil

**Keywords:** Online survey, COVID-19, Vaccination, Morbidity, Sequelae, Health situation, Brazil

## Abstract

**Introduction:**

The analysis of COVID-19 mortality revealed that the Brazilian population was critically impacted by the pandemic. However, many knowledge gaps remain regarding COVID-19 morbidity in the country. This article aims to analyze the consequences of the coronavirus disease on health situation of the Brazilian population.

**Methods:**

This was a cross-sectional epidemiological online survey using an electronic questionnaire between July and December 2023. The sampling method used was the virtual Respondent Driven Sampling (RDS). Changes in socioeconomic conditions were assessed in the post-pandemic period. Anti-COVID-19 vaccination coverage, prevalence of SARS-CoV-2 infection were estimated, as well as of sequelae lasting three months or more (Long COVID) among confirmed cases. Associations of Long COVID with self-reported heath status, sleep disorders, and depressive symptoms were analyzed.

**Results:**

The sample included 3805 individuals 18-years or older. Regarding vaccination, 61.5 % (95 % CI: 58.0 %–65.0 %) stated they had received 3‒4 doses. In the post-pandemic period, 41.6 % faced financial difficulties. Prevalence of confirmed SARS-CoV-2 infection was 40.2 %, 6.4 % of respondents reported having had COVID-19, although not confirmed by test, and 15.3 % did not know if they had been infected with the coronavirus. Among those infected, 32.0 % (95 % CI: 28.8 %–35.3 %) reported Long COVID, 21.4 % reported a COVID-19-related illness, and 5.2 % needed and obtained hospitalization. Long COVID was associated with worsening self-rated health, sleep disorders, feelings of depression and 27.7 % were unable to perform their usual activities for one month or more.

**Conclusion:**

The results of this study showed that Brazil was severely affected by the COVID-19 pandemic, both in terms of mortality and morbidity. The availability of timely post-pandemic data, as presented in this study, may be highly relevant to inform public policies aimed at promoting healthy behaviors, controlling NCDs, improving mental health care, and supporting specialized care for Long COVID within the public health system.

## Introduction

Studies on COVID-19 mortality have revealed that the Brazilian population has been severely affected by the pandemic. Inadequate management of the pandemic caused an unprecedented crisis in Brazil, highlighting the vulnerabilities of the health system in dealing with the emergency.^1^, ^2^, ^3^, ^4^, ^5^, ^6^, ^7^ The lack of equipment, hospital beds, ICU beds, and health care teams to address the growing demand undoubtedly exacerbated the lethality of the virus in Brazil, which was one and a half times higher than the global rate by the end of 2021.^8^

Further analyses of COVID-19 mortality showed the unequal impact of the pandemic by socioeconomic conditions.^9^^,^^10^ Mortality rates showed a decreasing gradient according to educational attainment, with illiterate individuals experiencing a mortality rate three times higher than those with higher education or more.^11^ Analysis at subnational levels also revealed significant regional differences, with higher mortality rates in the North and Central-West regions, particularly in the indigenous population, once again demonstrating Brazil’s pronounced educational, income, ethnical and regional disparities.^12^^,^^13^

The need for more knowledge about SARS-CoV-2 infection and other health issues directly or indirectly related to the coronavirus pandemic led to increased use of the internet as a rapid source of information.^14^ In Brazil, with the aim of investigating changes in the living and health conditions of the population during the COVID-19 pandemic, the “ConVid” – Behavior Survey was conducted in a virtual environment in 2020.^15^ Data were collected through chain sampling using the “virtual snowball” method.^16^

Findings from the ConVid – Behavior Survey conducted among the adult Brazilian population showed that the recommendation for home confinement^17^ had major socioeconomic implications, with a significant reduction in household income, especially among socially disadvantaged individuals.^18^ On the other hand, the restriction of physical contact, loss of freedom of movement, and uncertainties surrounding the disease triggered feelings of distress, anxiety, and sadness, leading to sleep problems, depression, and other mental health disorders.^19^, ^20^, ^21^ Similar findings were observed in surveys conducted in other countries.^22^, ^23^, ^24^, ^25^

Although it is widely recognized that the COVID-19 pandemic led to significant changes in Brazil’s mortality profile, with excess deaths during the pre-vaccination period, many knowledge gaps remain regarding COVID-19-related morbidity in the country. These include SARS-CoV-2 infection prevalence, sequelae in those who became ill, and COVID-19-related complications among individuals with non-communicable chronic diseases, all of which have generated new demands for healthcare and health promotion. Additionally, the emergence of long-term post-infection sequelae represents a new challenge for public health. After the initial infection with SARS-CoV-2, approximately 1 in 10 people experience COVID-19-related sequelae lasting three months or more, also known as Long COVID.^26^

To investigate these issues, a new survey was conducted between July and December 2023, called the “ConVid-2 – Behavior Survey”. A self-administered virtual questionnaire was used, accessible by mobile phone or computer with internet access. The Respondent Driven Sampling (RDS) method was used to collect information, starting with the distribution of a fixed number of invitations containing a link to the electronic questionnaire.^27^

Based on data collected in the ConVid-2 survey, this article aims to describe various aspects related to the COVID-19 pandemic that have affected the health situation of the Brazilian population, including vaccination coverage, SARS-CoV-2 infection prevalence, symptoms, disabilities, diseases or sequelae lasting three months or more (known as Long COVID), and required hospitalizations.

## Material and methods

### Study design

This is a cross-sectional epidemiological study conducted through chain sampling in a virtual environment among the adult Brazilian population (aged 18 and over) between July and December 2023. The project, titled “ConVid-2 – Behavior Survey”, was carried out by the Oswaldo Cruz Foundation (Fiocruz), in partnership with the Federal University of Minas Gerais (UFMG), the University of Campinas (Unicamp), the Federal University of Ouro Preto (UFOP), and the Federal University of Sergipe (UFS).

The inclusion criteria were being 18-years or older and residing in Brazil during the COVID-19 pandemic.

The “ConVid-2 Behavior Survey” project was approved by the National Research Ethics Committee (CONEP) on December 22, 2022, under protocol number 5.836.202.

### Peer recruitment

The chain sampling method used was Respondent Driven Sampling (RDS) in a virtual format, initiated by sending a fixed number of invitations containing the link to access the electronic questionnaire via the WhatsApp social network or email.

Recruitment connections between recruiters and their recruits were recorded to allow for statistical adjustment of sample weights and variance estimation based on the sampling design.^28^

To begin the information gathering phase via RDS, supporters (seeds) were selected in Brazil's 27 states through targeted selection. To ensure sample diversity, supporters with different sociodemographic characteristics were selected. The seeds sent the survey link to 24 people in their social networks residing in the same state. The link was sent to one individual in each stratum composed of sex, age group (18‒29; 30‒44; 45‒59; 60+), and level of education (incomplete elementary school; complete elementary school/incomplete high school; complete high school or higher).

The survey participants invited by the seeds constituted the first wave of the recruitment chain. They, in turn, invited 3 to 5 people from their social networks, comprising the second wave of the recruitment chain. This process continued successively, ultimately forming the sample of individuals recruited in a chain through virtual social networks. In the peer recruitment process, it was recommended that only one person per household respond to the questionnaire.

The minimum sample size of 3600 people aged 18 or older was calculated based on the estimate for a simple random sample and adjusted for a design effect of 2.^29^ Further details on the use of the RDS method in a virtual environment and on the information collection procedure were presented in another article from the ConVid-2 survey.^30^

### Variables

a) COVID-19 vaccination coverage – Measured by the proportion of individuals who reported receiving at least one dose of a COVID-19 vaccine, stratified by the number of doses.
b) SARS-CoV-2 infection prevalence – Measured by the proportion of individuals who reported having received a laboratory-confirmed positive COVID-19 diagnosis.
c) Socioeconomic conditions – Assessed through questions such as: How did the COVID-19 pandemic affect your employment/occupation? How did the pandemic affect your household income, considering the total income of all residents in your household? Before the pandemic, did your household experience financial difficulties? Currently, does your household experience financial difficulties?
d) Food insecurity – Assessed with the following questions: In the three months before the pandemic (December 2019 to February 2020), was there ever a concern in your household about not having enough money to buy food? In the past three months, was there ever a concern in your household about not having enough money to buy food?
e) COVID-19-related problems – Assessed through the following outcomes: interruption of usual activities for one month or more; development of a disease; worsening of self-rated health; hospitalization; sleep disturbances; symptoms, disabilities, illnesses, or sequelae related to COVID-19 that persisted for three months or longer (Long COVID).
f) Mental health issues: The questions referred to sleep problems in the last two weeks, self-reported medical diagnosis of depression, and the PHQ-9 (Patient Health Questionnaire-9) to assess degree of depression severity via questionnaire.^31^

## Results

Table 1 presents the results related to COVID-19 vaccination. From the 3805 participants, 29 (0.7 %) did not answer the question, 48 (1.3 %) did not take any dose of the vaccine, and 3728 (98.0 %) reported receiving at least one dose. The majority (61.5 %) took three to four doses. Only 1.3 % of participants reported not having received any dose, with the main reasons cited being *“not believing in the effectiveness of the vaccine”* and *“having already had COVID-19”.*

**Table 1:** COVID-19 Vaccination coverage, COVID-19 prevalence (confirmed or not), place of diagnosis, and hospitalization requirement. ConVid 2 – Behavioral Survey, 2023.

Variable		n	%	95 % CI
**How many doses of the COVID-19 vaccine have you received?**				
	Did not answer	29	0.7	0.3 – 1.9
	None	48	1.3	0.7 – 2.5
	1 ‒ 2 doses	689	18.1	15.8 – 20.6
	3 ‒ 4 doses	2341	61.5	58.0 – 65.0
	5 or more doses	698	18.4	15.2 – 22.0
	At least one dose	3728	98.0	96.6 – 98.9
**Have you ever had COVID-19?**				
	Did not answer	8	0.2	0.1 – 0.5
	No	1442	37.9	32.8 – 43.3
	Do not know	582	15.3	11.1 – 20.6
	Yes, but not confirmed by test	243	6.4	4.5 – 8.9
	Yes, confirmed by test	1530	40.2	34.2 – 46.5
**Where was COVID-19 confirmed?**				
	Pharmacy	156	10.2	8.2 – 12.5
	Primary Health Unit or Emergency Care (SUS)	736	48.2	40.7 – 55.7
	Hospital	201	13.1	9.8 – 17.4
	Private lab	267	17.4	14.2 – 21.2
	Self-test	88	5.7	4.2 – 7.8
	Other	82	5.4	3.7 – 7.7
**Needed and obtained hospitalization due to confirmed COVID-19**		80	5.2	3.1 – 8.6

Prevalence of SARS-CoV-2 infection confirmed by laboratory testing was 40.2 % (95 % CI: 34.2 %–46.5 %). Additionally, 6.4 % of respondents reported having had COVID-19, although not confirmed by test and 15.3 % did not know if they had been infected with the coronavirus, making it difficult to estimate precise prevalence of SARS-CoV-2 infection. For nearly half (48.2 %) of those people who had COVID-19 confirmed by laboratory testing, the diagnosis was obtained at primary health care units (UBS) or emergency care units (UPA) of the Unified Health System (SUS); 17.4 % were diagnosed in private laboratories, 15.9 % in pharmacies or via self-testing, and 13.1 % in hospitals. The percentage of individuals who needed and obtained hospitalization due to COVID-19 was 5.2 % (95 % CI: 3.1 %–8.6 %), as shown in Table 1.

Table 2 presents the socioeconomic conditions before and after the COVID-19 pandemic. Excluding individuals who had never worked, 14.1 % were left without work or income during the pandemic. A significant decrease in household income was also found: 26.3 % reported a slight decrease, and 18.0 % reported a substantial reduction. Before the pandemic, 31.0 % faced financial difficulties. After the pandemic, this proportion increased to 41.6 %. In relation to food insecurity, around 30 % of respondents reported concern about not having enough money to buy food both in the three months before the pandemic and in the three months prior to the survey.

**Table 2:** Socioeconomic conditions, financial difficulties, and food insecurity before and after the COVID-19 Pandemic^a^. ConVid 2 – Behavioral Survey, 2023.

Variable		n	%	95 % CI
**How did the COVID-19 pandemic affect your job/occupation?**				
	The participant did not work before and still does not	802	21.2	17.8 – 25.2
	Worked before and continued during the pandemic	1754	46.5	43.3 – 49.8
	Stopped working but kept receiving income	410	10.9	7.1 – 16.3
	Stopped working and lost income	532	14.1	11.0 – 17.9
	Started working during the pandemic	274	7.3	5.7 – 9.3
**How did the pandemic affect household income (sum of all residents)?**				
	Increased	201	5.3	4.1 – 6.8
	Remained the same	1818	47.9	44.0 – 51.9
	Decreased slightly	997	26.3	22.8 – 30.1
	Decreased significantly	683	18.0	15.3 – 21.0
	No income	93	2.5	1.6 – 3.7
**Before the pandemic, did your household face financial difficulties?**		1174	31.0	26.6 – 35.7
**Currently, does your household face financial difficulties?**		1574	41.6	36.7 – 46.7
**Concern about not having enough money to buy food:**				
	In the 3-months before the pandemic (Dec 2019 – Feb 2020)	1146	30.2	25.4 – 35.5
	In the last 3-months	1202	31.8	26.2 – 38.0

a Among participants who answered the question.

Regarding symptoms, disabilities, illnesses, or sequelae related to COVID-19 lasting three months or more (Long COVID), 32.0 % of participants reported such conditions and among individuals who had at least one Non-Communicable Disease (NCD), the percentage was 36.2 %. The main health problems cited included: fatigue, tiredness or weakness (58.0 %), memory loss or difficulty concentrating (48.4 %), hair loss (33.4 %), loss smell or taste (32.0 %), muscle or back pain (31.7 %), respiratory issues (30.0 %), persistent cough (29.3 %), and dizziness/vertigo (19.5 %). When asked whether they had developed a disease as a result of COVID-19 sequelae, 21.4 % answered affirmatively, and 27.7 % reported having stopped their usual activities for one month or more (Table 3).

**Table 3:** Health Consequences of COVID-19. ConVid 2 – Behavioral Survey, 2023.

Indicator		n	%	95 % CI
**At least one non-communicable disease (NCD) and lab confirmed COVID-19**		858	40.8	33.5 – 48.5
**Post-COVID symptoms lasting 3-months or more among confirmed cases (Long COVID)**		488	32.0	28.8 – 35.3
	Long COVID among those with at least one NCD	311	36.2	31.4 – 41.5
**Long COVID main reported symptoms**				
	Fatigue, tiredness or weakness	283	58.0	51.2 – 64.4
	Memory loss or difficulty concentrating	236	48.4	40.1 – 56.7
	Hair loss	163	33.4	25.8 – 41.9
	Loss of smell or taste	156	32.0	25.5 – 39.3
	Muscle or back pain	155	31.7	25.4 – 38.7
	Breathing problems	147	30.0	24.0 – 36.8
	Persistent cough	143	29.3	23.1 – 36.4
	Dizziness or vertigo	95	19.5	13.6 – 27.1
**Duration of symptoms**				
	3–6 months	218	44.7	36.9 – 52.9
	6–12 months	103	21.2	15.5 – 28.4
	12–24 months	75	15.4	11.2 – 20.9
	Over 24 months	91	18.6	14.4 – 23.5
**Unable to perform usual activities for 1-month or more due to symptoms**		135	27.7	20.1 – 37.0
**Developed a new illness as a result of COVID-19**		105	21.4	13.7 – 31.8

Health status consequences of Long COVID are presented in Table 4. Among all participants, 62.0 % rated their health as good or very good. However, among individuals with Long COVID, this percentage was significantly lower (47.3 %), while the proportions of self-rated health fair and poor/very poor health were higher than among those who did not have Long COVID. Across the total sample, 22.8 % reported having sleep problems almost every day. Among those with Long COVID, this figure rose to 32.9 % and 14.4 % took sleeping pills every day. The data also show that 14.7 % of respondents self-reported having depression, while among those with Long COVID, the proportion was higher at 22.8 %, and 20.3 % took antidepressants every day. According to PHQ9, severe depression was found in 6.9 % of participants who did not have Long COVID and 15.7 % of those who did have Long COVID.

**Table 4:** Association Between Long COVID and Health Perception, Chronic Disease, Sleep, and Depression. ConVid 2 – Behavioral Survey, 2023.

Indicator		No Long COVID		Long COVID		Total Sample	
		%	95 % CI	%	95 % CI	%	95 % CI
**Self-rated health**							
	Good/Very good	72.6	64.6 – 79.4	47.3	39.2 – 55.6	62.0	57.5 – 66.2
	Fair	25.6	19.2 – 33.2	45.7	38.6 – 52.9	33.2	29.4 – 37.3
	Poor/Very poor	1.8	1.1 – 3.0	7.0	3.6 – 13.1	4.8	3.2 – 7.1
**Sleep problems nearly every day**							
	No	82.2	77.9 – 85.8	67.1	61.0 – 72.6	77.2	72.9 – 81.0
	Yes	17.8	14.2 – 22.1	32.9	27.4 – 39.0	22.8	19.0 – 27.1
**Used sleep medication in the last 2-weeks**							
	Daily	7.8	5.3 – 11.4	14.4	8.6 – 23.2	11.2	8.7 – 14.1
	Occasionally	11.6	8.6 – 15.4	13.9	9.3 – 20.1	12.7	8.6 – 18.4
	None	80.6	77.1 – 83.6	71.7	62.0 – 79.7	76.1	71.1 – 80.5
**Diagnosed with depression**							
	No	86.6	83.2 – 89.4	77.2	70.7 – 82.5	85.3	82.9 – 87.4
	Yes	13.4	10.6 – 16.8	22.8	17.5 – 29.3	14.7	12.6 – 17.1
**Severity of depression (PHQ9)**							
	None	38.8	33.4 – 44.6	24.2	16.8 – 33.5	36.5	29.9 – 38.6
	Very little	25.1	21.0 – 29.8	24.8	18.6 –32.3	23.8	21.6 – 28.8
	Moderate	18.3	14.1 – 23.5	17.3	13.7 – 21.7	16.2	14.7 – 21.8
	Moderately severe	10.9	7.7 – 15.0	18.0	12.6 – 24.9	14.8	10.7 – 16.1
	Severe	6.9	4.7 – 9.9	15.7	11.5 – 21.1	8.7	7.8 – 12.1
**Used antidepressants in the last 2-weeks**							
	Daily	13.0	10.0 – 16.8	20.3	14.4 – 27.9	13.5	11.5 – 15.8
	Occasionally	2.4	1.5 – 3.7	3.2	1.9 – 5.2	6.0	3.1 – 11.4
	None	84.6	81.1 – 87.6	76.5	69.4 – 82.4	80.5	76.4 – 84.1

## Discussion

The results from the second phase of the ConVid – Behavior Survey, conducted among the adult Brazilian population three years after the onset of the COVID-19 pandemic, revealed significant losses in employment and income, an increase in the proportion of people experiencing financial difficulties, and a worsening of social inequalities. As shown in the 2020 survey, the COVID-19 pandemic led to major reductions in household income and employment status,^18^ which likely have not yet been fully recovered.

This second phase of the survey enabled the investigation of vaccination coverage, COVID-19 prevalence (with or without laboratory confirmation), and disease-related complications. Vaccination coverage with at least one dose was high 98.0 %, close to that found (96.6 %) in the Continuous National Household Sample Survey (PNAD COVID-19, 2023) among people aged 18 and over.^32^ This survey was conducted through a partnership between the Ministry of Health and the Brazilian Institute of Geography and Statistics (IBGE). In Brazil, vaccination policy falls under the responsibility of the National Immunization Program (PNI) of the Ministry of Health. Through this program, the federal government provides various immunobiological products through the Unified Health System (SUS) free of charge, including COVID-19 vaccines since 2021. In February 2022, Brazil consolidated its independent COVID-19 vaccine production program, ensuring the delivery of sufficient doses to meet the 2022 demands and sustain mass vaccination, especially with the emergence of new SARS-CoV-2 variants.^33^

It is important to note that at least two doses of the vaccine are required to achieve the minimum effective regimen. This target was reached by 93.6 % of ConVid-2 survey participants. Similar results were observed in the COVID-19 module of the 2023 PNAD, with 92.3 % of individuals aged 18-years and older having received two or more doses.^32^ One possible explanation for the slightly higher vaccination coverage observed in the ConVid-2 survey lies in the composition of the study sample. In online surveys, individuals without internet access, with lower levels of education, or who are very elderly face substantial barriers to responding to the questionnaire and are therefore underrepresented.^15^

A previous study demonstrated the existence of socioeconomic inequalities in vaccination coverage.^34^ The proportion of individuals who had received four or more vaccine doses was significantly lower among those with a household per capita income below half the minimum wage (40.6 %) compared with those with higher per capita income (61.2 %). Significant differences were also observed among residents of households experiencing financial hardship (45.3 %) and food insecurity (44.2 %), compared with those not facing these conditions (53.1 % and 52.4 %, respectively).

Approximately 40 % of Brazilians reported a confirmed COVID-19 diagnosis, while 6 % reported having had COVID-19 without laboratory confirmation. This prevalence estimate is similar to that reported by PNAD COVID-19.^32^ The total number of SARS-CoV-2 infections among individuals aged 5-years or older was estimated at 68.8 million. Assuming the same proportional age distribution of confirmed cases (5–17 and ≥ 18-years) for the total number of reported cases (confirmed or self-reported), the PNAD estimated prevalence was 45.7 %.

Furthermore, 15.3 % of the survey participants did not know whether they had been infected with the coronavirus, likely due to limited access to testing within the public health system.^4^ The results of this study showed that fewer than half of the respondents underwent testing in public primary care units probably because months after the arrival of the COVID-19 epidemic in Brazil, diagnostic testing supplies in public health services were still unavailable.^5^

Among those with confirmed COVID-19, 5.2 % needed and obtained hospitalization, overwhelming hospitals and intensive care units. A study revealed high COVID-19 fatality rates in Brazilian hospitals, which could have been avoided if there had not been intense pressure on health services.^35^ The results of the PNAD COVID survey^32^ showed a slightly lower hospitalization rate, 4.2 %, probably because the estimates were calculated for the population aged 5-years and older. Similarly, the Epicovid study showed a hospitalization rate of 4.5 % for the total population.^36^

Long COVID, as defined by the World Health Organization (WHO), refers to symptoms and sequelae persisting for three months or more after acute SARS-CoV-2 infection.^37^ In this study, Long COVID was identified in 32.0 % of those with confirmed infections ‒ equivalent to 12.8 % of all participants, close to the global estimate of 10 %.^26^ Long COVID had significant consequences for Brazilians’ health. Self-perceived health was worse among those with Long COVID. Additionally, sleep problems and depressive symptoms were more prevalent. According to the 2019 National Health Survey, 9.9 % of Brazilians had been medically diagnosed with depression,^38^ a figure lower than the 14.7 % observed in this 2023 study. Among people with Long COVID, this proportion increased to 22.8 %. Comparisons between self-reported depression diagnoses and PHQ-9 scores indicated that severe depression was also substantially more frequent among participants with Long COVID.

The main Long COVID-related health problems reported in Brazil were like those found in other countries. A study conducted in Japan revealed similar prolonged COVID symptoms and their association with reduced health-related quality of life. Symptoms such as dyspnea, fatigue, headache, and muscle weakness were linked to worse scores in physical health, while poor concentration, sleep disturbances, fatigue, and headache were associated with poorer mental health.^39^

Another study in Brazil also evidenced the country is experiencing significant levels of health issues related to Long COVID, with chronic conditions persisting after the initial SARS-CoV-2 infection, which may place a burden on individuals, impacting their employment and socioeconomic status.^40^ In addition to reduced household income and food insecurity, this study revealed that nearly 28 % of individuals with Long COVID were unable to perform their usual activities for one month or more. These findings highlight the need for dedicated care strategies to ensure timely diagnosis and management of Long COVID within the public health system (SUS).^41^ Moreover, these individuals may require special clinical attention to improve mental health and prevent the worsening of anxiety and depression symptoms, sleep disturbances, and persistent fatigue.^42^

In addition to pre-existing challenges in reducing premature mortality from chronic diseases, the health crisis caused by COVID-19 appears to have led to disrupted access to healthcare services during the period of social restrictions measures. As a result, the monitoring and care of individuals with NCDs were compromised, contributing to the deterioration of key NCD control indicators in the country.^43^^,^^44^

Among the limitations of this study are those related to chain sampling in online surveys. Individuals without internet access have zero probability of selection; those with lower levels of education and older adults often face difficulties in completing online questionnaires, which can interrupt recruitment chains; and selection probabilities and nonresponse rates cannot be estimated, as participation is voluntary. To obtain a representative sample of the Brazilian population, a post-stratification procedure was applied using population estimates from the 2022 PNAD, but the non-inclusion of a variable associated with an outcome during the post-stratification process may result in biased estimates. Besides the specific limitations of online surveys, all results were self-reported, increasing susceptibility to recall and reporting bias as most national health surveys. However, comparisons of vaccination coverage, COVID-19 prevalence, and hospitalization rates demonstrated consistency with the results of national face-to-face surveys, supporting the validity of the estimates.

## Conclusion

Among individuals who reported Long COVID, certain health problems persist to this day, such as worsened self-perception of health, sleep disturbances, and depressive symptoms. In this context, the availability of timely post-pandemic data, as presented in this study, may be highly relevant to inform public policies aimed at promoting healthy behaviors, controlling NCDs, improving mental health care, and supporting specialized care for Long COVID management within the public health system. Longitudinal follow-up and clinical validation of Long COVID would be very important in strengthening our knowledge about the disease sequelae over time.

## Funding

This work was supported by the Departamento de Ciência e Tecnologia (DECIT) do Ministério da Saúde do Brasil (Processo: 25000.156338/2022-60).

## Data availability statement

The data that support the findings of this study are available from the corresponding author upon reasonable request.

## Conflicts of interest

The authors declare no conflicts of interest.
